# CD8+ TCR Transgenic Strains Expressing Public versus Private TCR Targeting the Respiratory Syncytial Virus K^d^M2_82–90_ Epitope Demonstrate Similar Functional Profiles

**DOI:** 10.1371/journal.pone.0099249

**Published:** 2014-06-04

**Authors:** Erez Bar-Haim, Noam Erez, Allison M. W. Malloy, Barney S. Graham, Tracy J. Ruckwardt

**Affiliations:** 1 Vaccine Research Center, National Institute of Allergy and Infectious Disease, National Institutes of Health, Bethesda, Maryland, United States of America; 2 Department of Biochemistry and Molecular Genetics, Israel Institute for Biological Research, Ness-Ziona, Israel; 3 Department of Infectious Diseases, Israel Institute for Biological Research, Ness-Ziona, Israel; University of Iowa, United States of America

## Abstract

Our previous work has characterized the functional and clonotypic features of two respiratory syncytial virus (RSV) epitope-specific T cell responses in mice. Following single-cell sequencing, we selected T cell receptor sequences to represent both a public and a private clone specific for the dominant K^d^M2_82–90_ epitope for the generation of T cell receptor transgenic (TCR Tg) mice. We evaluated cells from these TCR Tg strains for three major functions of CD8+ T cells: proliferation, cytokine production and cytolytic activity. *In vitro* comparisons of the functional characteristics of T cells from the newly-generated mice demonstrated many similarities in their responsiveness to cognate antigen stimulation. Cells from both TRBV13-1 (private) and TRBV13-2 (public) TCR Tg mice had similar affinity, and proliferated similarly *in vitro* in response to cognate antigen stimulation. When transferred to BALB/c mice, cells from both strains demonstrated extensive proliferation in mediastinal lymph nodes following RSV infection, with TRBV13-2 demonstrating better *in vivo* proliferation. Both strains similarly expressed cytokines and chemokines following stimulation, and had similar Granzyme B and perforin expression, however cells expressing TRBV13-1 demonstrated better cytolytic activity than TRBV13-2 cells. These new, well-characterized mouse strains provide new opportunities to study molecular mechanisms that control the phenotype and function of CD8+ T cell responses.

## Introduction

Three decades of work on the murine model of human respiratory syncytial virus has been useful for defining several aspects of basic T cell biology [1], [2]. These include the role of CD8+ T cells in viral clearance and immunopathology [3], [4], the importance of CD8+ T cells in influencing CD4+ T cell function [5], and the influence of vaccine priming and allergic inflammation on CD4+ T cell differentiation and pathology post-infection [6]–[8]. More recently, this model has been used to determine mechanisms of T cell regulation of immune responses to infection [9]–[12], and been used to demonstrate age-dependent differences in CD8+ T cell responses [13] and modulation by prostanoids and leukotrienes [14]. The murine model of RSV is therefore uniquely positioned to address questions at the intersection of viral infection and allergic inflammation and determine how interactions between virus and host affect viral clearance, lung pathology and airway physiology. This distinguishes it from other well-established murine models of viral infection commonly used for studying T cell biology such as LCMV, influenza, or MCMV. Here we describe the design and functional characterization of two novel T cell receptor transgenic mice with CD8+ T cells specific for the K^d^M2_82–90_ epitope that provide the field with unique opportunities to simultaneously study the response of different clones during infection and understand the regulation and effector function of T cells in the context of an acute respiratory virus infection.

RSV infection in BALB/c mice induces a strongly dominant response to the M2 protein-derived SYIGSINNI peptide bound to H-2K^d^. CD8+ T cell responses to this epitope have been found to contribute to both viral clearance and immunopathology following RSV infection of BALB/c mice [4], [15]–[18]. We have further evaluated responses to this epitope in CB6F1 mice, which can generate responses to both H-2d and H-2b-restricted peptides. While the K^d^M2_82–90_ response remains dominant in CB6F1, the dominance of the response to this epitope cannot be simply explained by difference in the precursor frequency for these epitopes, as both responses were found to have a similar number of precursor cells [13]. Based on our observations about the functional profile of epitope-specific responses in RSV-infected CB6F1 mice, the K^d^M2_82–90_-specific response is more inflammatory and responsible for significant immunopathology, as manifested by weight loss in infected mice. Cells specific for this epitope also often lack functionality with regard to cytokine production and cytotoxicity *in vivo* [18], [19]. The cytotoxic activity of the subdominant D^b^M_187–195_-specific T cell response is higher than that of K^d^M2_82–90_-specific response, resulting in better viral clearance and less illness in mice with a dampened K^d^M2_82–90_-specific response [18].

We have analyzed the clonotype of CD8+ T cells to the two major RSV epitopes, K^d^M2_82–90_ (M2) and D^b^M_187–195_ (M), following RSV infection in CB6F1/J mice [20]. We found that M2-specific TCRs were largely public and almost exclusively utilized TRBV13-2*01. We used our single-cell clonotype data to guide T cell receptor selection for the generation of two distinct TCR transgenic mouse strains (TCR Tg) specific for the dominant M2 epitope, a method for TCR Tg mouse generation that has not been previously used. Paired alpha and beta sequences representing a TRBV13.2-expressing, public TCR that is most characteristic of the M2-specific response and paired sequences representing a more private, TRBV13-expressing clone were selected for the generation of TCR Tg mice. We present here the initial characterization of these two TCR Tg strains specific for the same viral epitope. We compare the functional profile following stimulation with a range of peptide concentrations. The two lines demonstrated a relatively similar profile both *in vitro*, and *in vivo* following RSV infection, with TRBV13-2 demonstrating more proliferation in vivo following infection and TRBV13-1 demonstrating better CTL activity at low concentrations of peptide. These new strains of TCR Tg mice will be useful for gaining a better understanding of CD8+ T cell responses to RSV infection.

## Materials and Methods

### Ethics statement

All mice used in this study and analysis were maintained according to the guidelines of the NIH Guide to the Care and Use of Laboratory Animals and the approval of the Animal Care and Use Committee of the Vaccine Research Center (VRC), National Institute of Allergy and Infectious Diseases at the National Institutes of Health. All mice were housed in a facility fully accredited by the Association for Assessment and Accreditation of Laboratory Animal Care International (AAALAC). All procedures were approved under animal care and use protocol numbers VRC-10-315, VRC-11-345, and VRC-14-466 and were conducted in strict accordance with all relevant federal and National Institutes of Health guidelines and regulations.

### Mice

Thy1.1^+^ CB6F1 mice were bred in house by crossing Thy1.1^+^ BALB/c mice (a generous gift from Jon Yewdell) with Thy1.1^+^ C57BL/6 (Jackson Labs, Bar Harbor, ME or bred in-house). The TCR transgenic mice (designated TRBV13.1 and TRBV13.2) were produced by NCI-Frederick Laboratory Animal Sciences Program (LASP) transgenic mouse model service, and subsequently bred as heterozygotes in house. Both strains were generated on a BALB/c background by microinjection with two constructs, one containing the full-length cDNA of the α gene and one with the β gene as elaborated in the Results (see Table 1).

**Table 1:** Description of TCR Tg strains.

Name	Specificity	TRBV	CDR3β	TRAV	CDR3α	clonotype	Mouse strain
**TRBV13-2**	K^d^M2_82–90_	TRBV13-2*01	CASGAGTGYAEQFF	TRAV7-3*01	CAVNSGYNKLTF	Public	**BALB/c**
**TRBV13-1**	K^d^M2_82–90_	TRBV13-1*02	CASSDGGKEVFF	TRAV12-2*01	CALRNNNNAPRF	Private	**BALB/c**

### Synthetic peptide

RSV M2_82–90_ (SYIGSINNI) peptide was derived from the RSV M2 protein. The peptide was synthesized by Anaspec, Inc. (San Jose, CA), and confirmed to be >95% pure by analytical high-performance liquid chromatography at the NIAID peptide core facility (Bethesda, MD).

### Lymphocyte stimulation

For the majority of experiments the cells were processed as follows. Splenocytes were harvested from naïve TCR Tg mice and brought into a single cell suspension by tissue dissociation using a GentleMACS machine (Miltenyi). Lymphocytes were purified using Fico-LITE (Atlanta Biologicals), washed, and resuspended in lymphocyte medium (RPMI, 10%FBS, 2 mM Glutamine, 1 mM Sodium Pyruvate, non-essential amino acids, 25 mM HEPES, 5×10^−5^ M β-mercaptoethanol and Pen/Strep antibiotics). Lymphocytes were stimulated with peptide at concentrations ranging from 1×10^−6^ M to 1×10^−12^ M, in the presence of 1 µg/ml of costimulatory antibodies for CD28 and CD49d (BD Biosciences) and 10 U/mL IL-2 (PeproTech, NJ) unless stated otherwise. The stimulation was carried out in the presence of feeder cells: 5000 rad-irradiated CB6F1 Thy1.1+ splenocytes in a ratio of 1∶1 with the stimulated cells. All stimulations were carried out in 37°C, 5% CO_2_ humidified incubator for the indicated time points.

### Flow Cytometry

All washing procedures were done using flow buffer composed of PBS, 2%FBS and 0.05% NaN_3_. The following mAb clones were used for staining: CD3 (145-2C11), CD8 (2.43), IFNγ (XMG1.2), IL-2 (JES6-5H4), TNFα (MP6-XT22), Thy1.2 (53–2.1), CD69 (H1.2F3), CD25 (7D4), CD28 (37.51), PD1 (J43), CD107a (1D4B) CD107b (ABL-93), GranzymeB (GB11), Perforin (OMAK-D) and TCR β chain (H57-597). All antibodies were purchased from eBioscience (San Diego, CA), BD Biosciences (San Diego, CA) or Biolegend (San Diego, CA). For tetramer analysis, cells were surface stained with K^d^M2_82–90_ conjugated to PE (Beckman Coulter, San Diego, CA).

For proliferation analysis, splenocytes were labeled prior to stimulation with 5 µM carboxy-fluorescein diacetate succinimidyl ester (CFSE, Invitrogen) for 5 minutes at room temperature and washed 3 times with RPMI+10% FBS. For intracellular staining, cells were incubated for 5 hours in the presence of Golgi-Stop and Golgi-Plug (BD Biosciences) as directed, washed and surface stained. The cells were then fixed and permeabilized using a Cytofix/Cytoperm kit (BD Biosciences) according to the manufacturer's instructions prior to intracellular staining. For DNA content analysis, splenocytes were stimulated for 18 hours and fixed in cold 70% Ethanol. Cells were later stained for CD3 and CD8, washed and resuspended in propidium iodide (PI) solution (25 µg/ml in flow buffer) prior to acquisition. Samples were collected on an LSR-II flow cytometer (BD Biosciences, San Jose, CA) and data were analyzed using FlowJo version 9.5 (Tree Star, San Carlos, CA). All the analyses were performed on live, single cells. Viability was determined by staining with VIVID or Aqua (Life Technologies) following a singlet gate to identify single cells.

### *In vitro* CTL assay

Target CB6F1 splenocytes were harvested from naïve CB6F1, purified as described above and labelled with either 5 µM (high) or 0.25 µM (low) CFSE followed by 3 washes with lymphocyte medium. CFSE^high^ labeled cells were loaded with K^d^M2_82–90_ at desired concentration and CFSE^low^ labeled cells were loaded with 10^−6^ M K^d^–binding influenza virus A/Puerto Rico/8/34 NP_147–155_ (TYQRTRALV) peptide as internal control. CFSE^high^ and CFSE^low^ target cells were washed 3 times and mixed at 1∶1 ratio. Activated effector CD8+ T cells were isolated from TRBV13-1 and TRBV13-2 mice that were intraperitoneal injected 3 days earlier with 1×10^7^ PFU RSV A2. Effector cells were purified using a CD8+ T cell isolation kit (Miltenyi) for untouched isolation, counted and mixed with target cells at different effector to target (E∶T) ratios and incubated at 37°C. After 4 hours incubation, cells were washed twice in flow buffer and fixed with 3% paraformaldehyde. CFSE^high^ and CFSE^low^ labeled cells were counted by flow cytometry and specific cell lysis was calculated using the following formula: % specific lysis  =  [1-(ratioX/ratio control)] × 100 where ratioX is CFSEhigh/CFSElow in each sample and ratio control is CFSEhigh/CFSElow of control target cells that were incubated without effector cells.

### Adoptive transfers of TCR Tg CD8+ T cells and RSV infection

CD8+ T cell transgenic T cells were isolated from TRBV13-1 or TRBV13-2 mice using a CD8+ T cell isolation kit (Miltenyi) for untouched isolation, and labeled with 5 µM CFSE prior to intravenous (iv) transfer into recipient BALB/c mice. 5×10^6^ cells were transferred iv into each recipient one day prior to intranasal (in) infection with 2×10^6^ PFU of RSV A2 generated as previously described [21]. Four days after infection, mediastinal lymph nodes (MLN) were isolated to assess proliferation of the transferred CD8+ T cells. The percent of the original population that divided was calculated using the proliferation analysis module within the Flowjo software.

### Tetramer dilution/TCR affinity assay

Single-cell suspensions of splenocytes were stained with serially-diluted concentrations of PE-conjugated tetramers specific for K^d^M2_82–90_. All washing and staining procedures were performed in flow buffer. The highest tetramer concentration was defined as the minimal concentration that resulted in complete staining and separation of TCR Tg cells. The splenocytes were stained with 1∶2 serially diluted tetramers, and Mean Fluorescence intensity (MFI) was determined using FlowJo software.

### Secreted cytokine analysis

Splenocytes from the TCR Tg mice were stimulated as described above for 3 days. Supernatants were collected and immediately frozen. Frozen samples were shipped to Aushon Biosystems (Billerica, MA) for multiplex cytokine analysis.

### Statistical analysis

Statistical analysis was performed using one-way or two-way analysis of variance (ANOVA) and recommended post-tests in GraphPad Prism version 5.00 for Windows, GraphPad Software, San Diego California, USA, www.graphpad.com.

## Results

### Generation of TCR Tg mice

We previously described the single-cell TCR clonotypic analysis of tetramer-sorted M2- and M-specific CD8+ T cells from RSV-infected mice [20]. We focused on the dominant, M2-specific response and selected both a public and a private CDR3β sequence. TCR β chain cDNA (full-length, fully recombined) sequences were cloned into a transgenic vector that exploits the H-2K^b^ promoter and an Ig enhancer, and a pairing TCR α chain cDNA sequence was cloned into a CD2-based expression vector as previously described [22]. Proper pairing was assured based on the fact that both α and β chains were sequenced from a single sorted cell. The resulting two TCR Tg strains, both generated on a BALB/c background, are designated by their β chain variable gene usage as either TRBV13-1 or TRBV13-2 and are described in Table 1. All mice were maintained as heterozygotes by serial back-crossing of transgene-positive mice to wild-type mice, suggesting the transgenes are present at a single insertion site. As expected, approximately 50% of offspring routinely screened positive following staining of whole blood cells with antibodies specific for CD3, CD8, and the transgenic Vβ.

### TRBV13-1 and TRBV13-2 cells have similar affinity and V*β* expression

**Figure 1:**
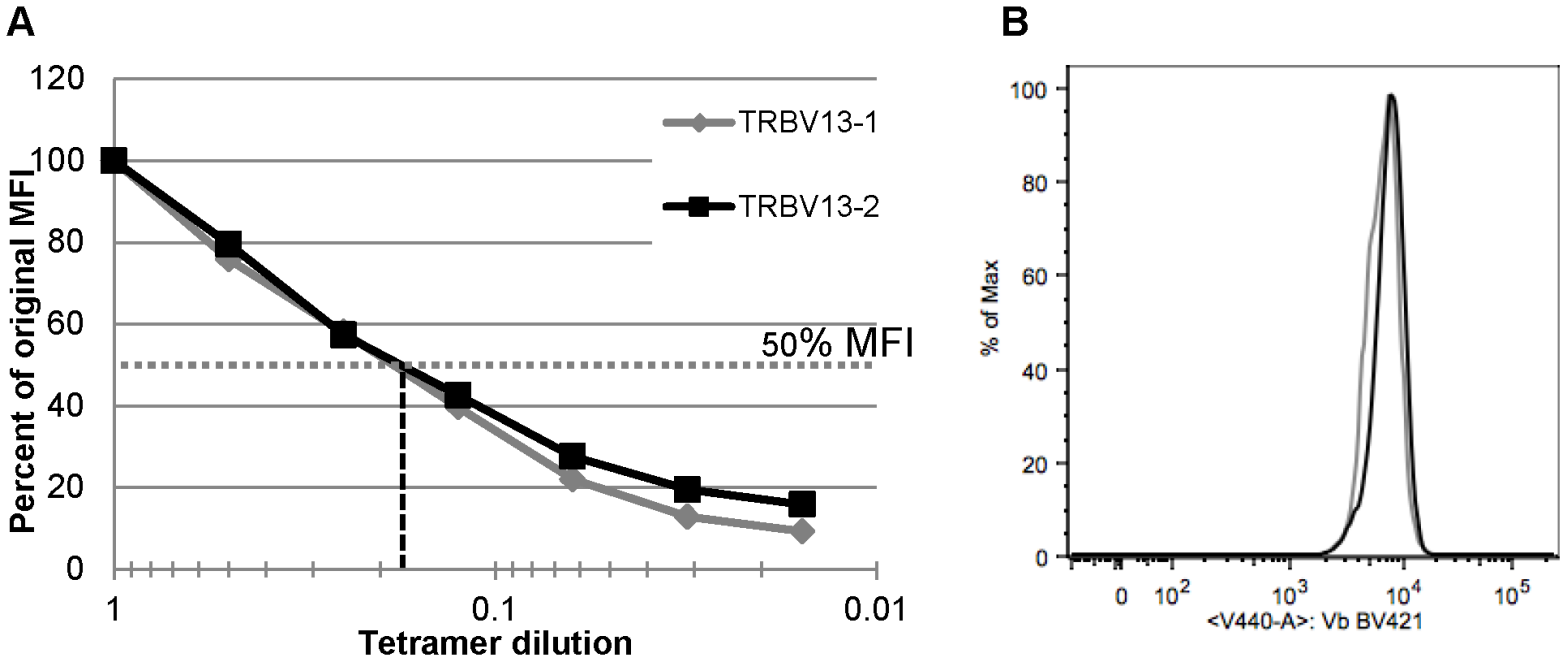
Tetramer dilution assay. (A) Splenocytes from TCR Tg mice were processed and stained with serially (1∶2) diluted PE-conjugated K^d^M2_82–90_ tetramer. Data are presented as the percentage of original MFI in CD3+ CD8+ cells stained with non-diluted tetramer. Data are representative of 3 independent experiments 1–2 mice in each. (B) Vβ expression was measured on CD8+ T cells from the blood of TRBV13-1 and TRBV13-2 TCR Tg strains.

The intrinsic affinity of each TCR clone was analyzed by staining with serial dilutions of tetramer at 4°C as previously described [23] (Figure 1A). The maximal tetramer concentration was determined by titration to be the lowest tetramer concentration that provided complete staining and separation of the tetramer-positive population of cells. Cells from both TRBV13-1 and TRBV13-2 strains demonstrated a similar half-maximal tetramer binding by mean fluorescence intensity (MFI), indicating a similar affinity for the K^d^M2_82–90_ epitope. We additionally measured surface Vβ expression by staining transgenic CD8+ T cells with a pan-Vβ antibody (clone H57-597), and expression was similar on the surface of CD8+ T cells from both strains (Figure 1B).

### TRBV13-1 and TRBV13-2 cells proliferate following cognate antigen stimulation *in vitro*, or RSV infection *in vivo*

**Figure 2:**
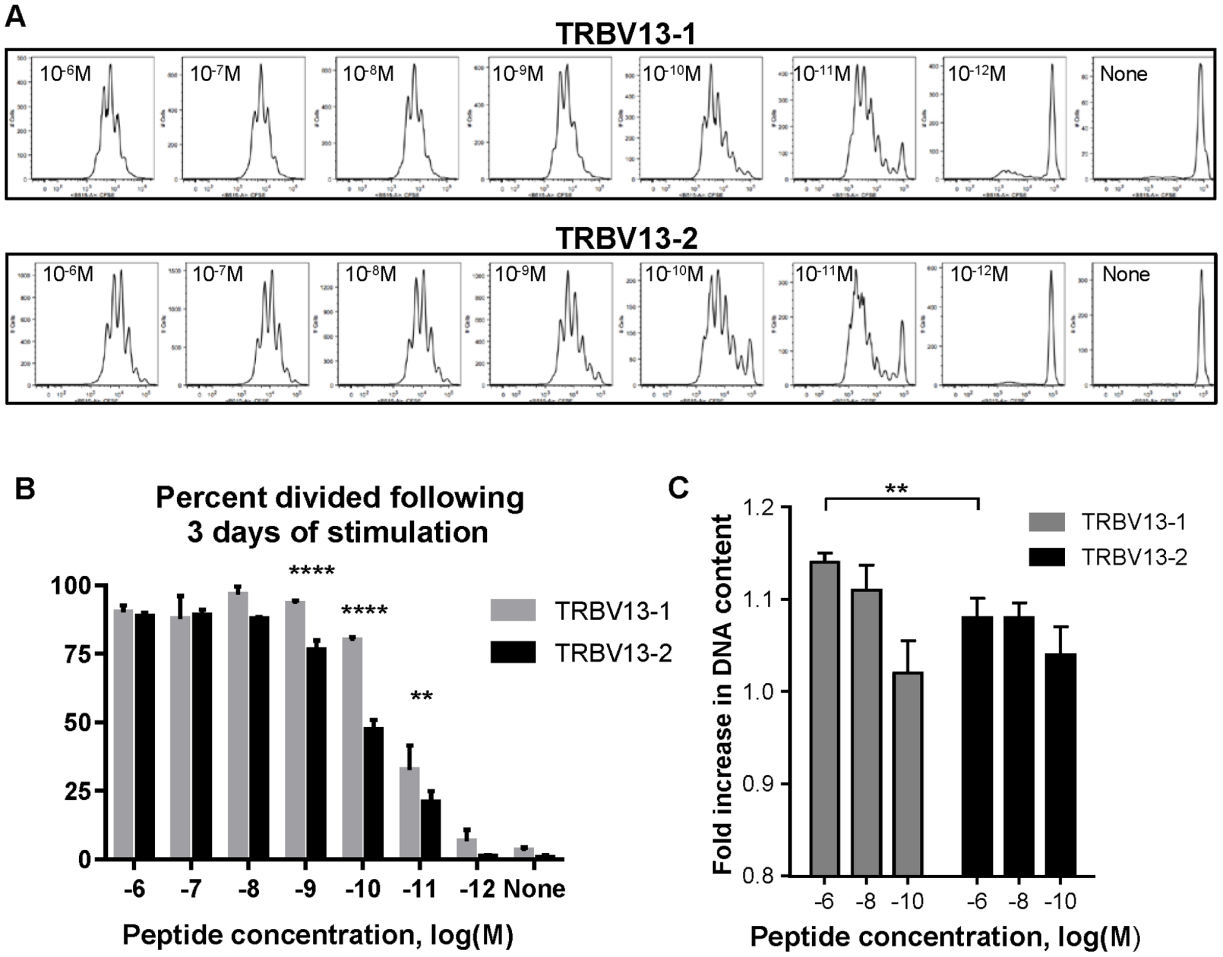
Proliferation of stimulated TCR Tg cells *in vitro*. (**A**) CFSE-labeled cells were stimulated in the presence of a range of peptide concentrations. Plots of CFSE, gated on live CD3+ CD8+ cells, are shown following 3 days of stimulation. (**B**) The percentage of cells from the original population that divided for TRBV13-1 and TRBV13-2 cells. Data are representative of 3 independent experiments, 1–2 mice in each, **p<0.01, ****p<0.0001. (C) Cells were stimulated for 18 hours, and their DNA content was determined by PI staining. Data are presented as fold DNA content in CD3+ CD8+ cells as compared to non-stimulated cells based on MFI of the PI staining. Data are calculated from 3–4 independent experiments, 1–2 mice in each, **p≤0.01.

Our prior analysis of precursor frequency for the M2- and M-epitope specific responses indicated that they have a similar precursor frequency in naïve, adult CB6F1 mice [13]. Despite similar numbers of precursors, the K^d^M2_82–90_ response rises rapidly after infection to significantly dominate the D^b^M_187–195_ response. We sought to address the proliferative capacity of our TRBV13-1 and TRBV13-2 clones using CFSE dilution analysis. TCR Tg T cells were stimulated by congenic Thy1.1 feeder cells from CB6F1 mice in the presence of decreasing M2 peptide concentrations ranging from 10^−6^ M to 10^−12^ M and in the presence of costimulatory antibodies (αCD28 and αCD49d) and IL-2 (10 U/mL). Cells were collected for analysis three days later. Both TRBV13-1 and TRBV13-2 demonstrated a threshold peptide concentration of 10^−11^ M, with no significant difference between 10^−12^ M and no peptide (Figure 2A). We measured the percentage of divided cells, i.e. the fraction of the original population that underwent proliferation (Figure 2B) for both TCR Tg lines. For both, most cells divided at peptide concentrations between 10^−6^ and 10^−10^ M before dropping off at the threshold concentration of 10^−11^ M. A higher frequency of TRBV13-1 cells proliferated at peptide concentrations between 10^−9^ and 10^−11^. Proliferation data from time points between 38 and 72 hours was exploited for doubling time analysis of each TCR Tg T cell population, and we calculated doubling times for dividing cells to be between 25–30 hours with no significant difference between the two TCR Tg cells (data not shown). In order to assess early responses of the TCR Tg clones, we stimulated the cells for 18 hours and looked at DNA synthesis by analyzing DNA content with PI staining (Figure 2C). DNA synthesis was observed for both TRBV13.1 and TRBV13.2 TCR Tg cells by 18 hours. DNA synthesis by TRBV13.1 cells was significantly higher than that of TRBV13.2 cells at high peptide concentrations, in spite of the same TCR specificity and comparable TCR affinity.

**Figure 3:**
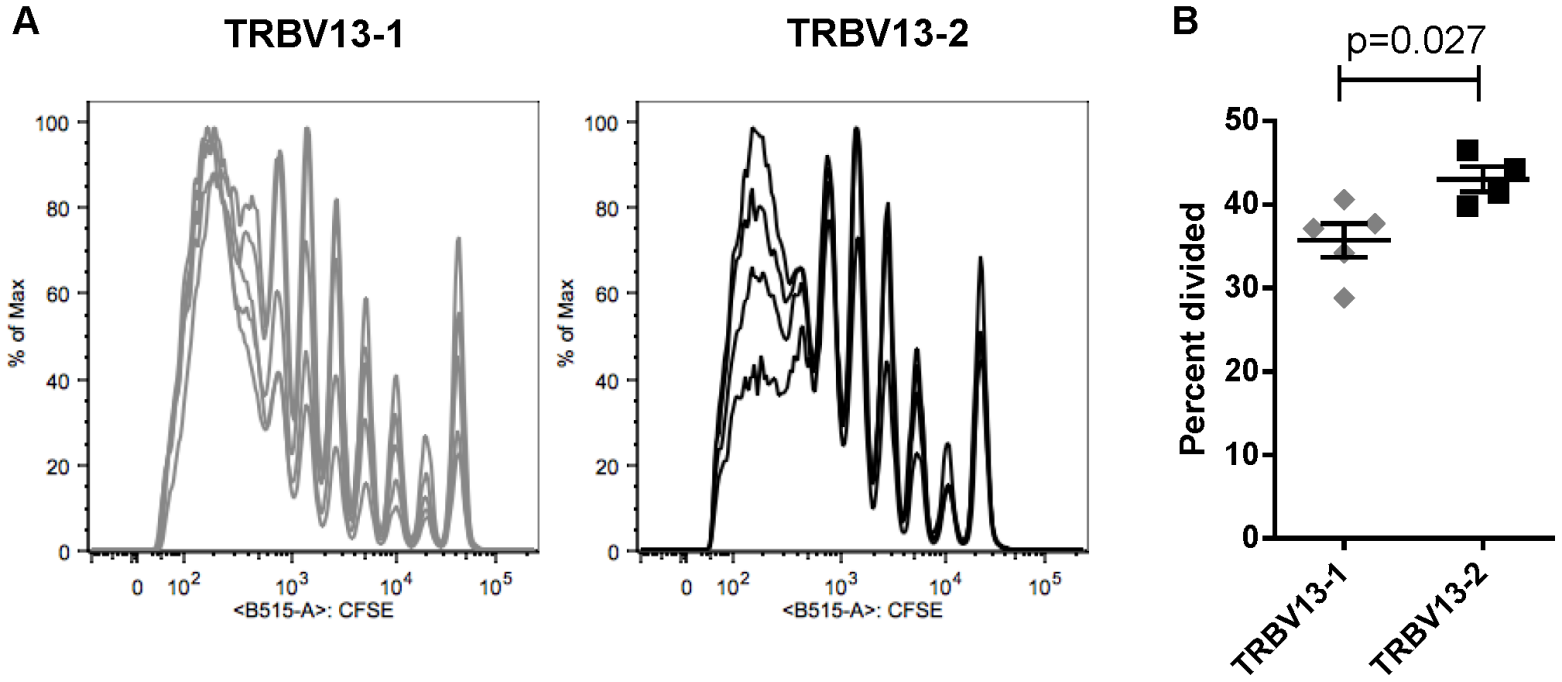
Proliferation of TCR Tg cells *in vivo* in RSV-infected BALB/c mice. (A) 5×10^6^ CFSE-labeled TRBV13-1 or TRBV13-2 cells were transferred in to naïve BALB/c recipients one day prior to in infection with 2×10^6^PFU of RSV A2. Four days after infection, CFSE dilution was measured in CD8+ T cells isolated from the mediastinal lymph node. (B) The percent of the original population that divided after 4 days in vitro was measured using FlowJo software.

We measured the ability of both TRBV13-1 and TRBV13-2 clones to respond to infection with live RSV virus by transferring 5×10^6^ CFSE-labeled CD8+ T cells from each strain into naïve BALB/c mice one day prior to infection with RSV. Four days after infection, we harvested mediastinal lymph nodes (MLN) from transferred mice, isolated and stained mononuclear cells and analyzed CFSE intensity in CD8+ T cells. Cells from both strains were highly proliferative in infected mice (Figure 3A). Calculation of the percent of the original population that had divided revealed that TRBV13-2 cells proliferated significantly better than TRBV13-1 cells *in vivo* (Figure 3B).

### Public and private M2-specfic T cells have similar cytokine and chemokine production following cognate antigen stimulation

**Figure 4:**
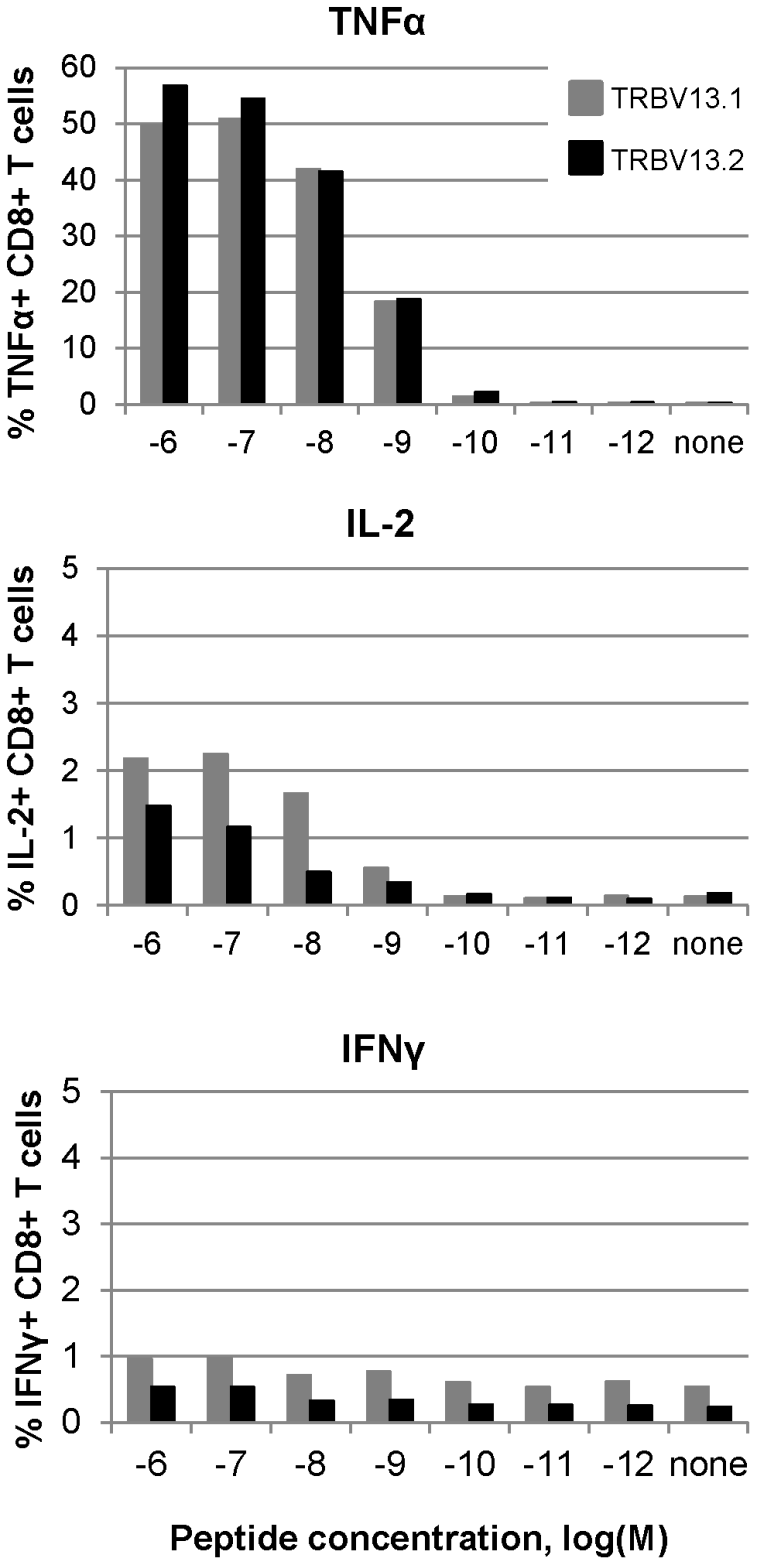
Cytokine production following 5 hour stimulation of TCR Tg cells. Cells were stimulated in the presence of specific peptide in graded concentrations for 5+CD8+ cells expressing each cytokine was determined by intracellular cytokine staining. Data are representative of two independent experiments, 1–2 mice in each.

**Figure 5:**
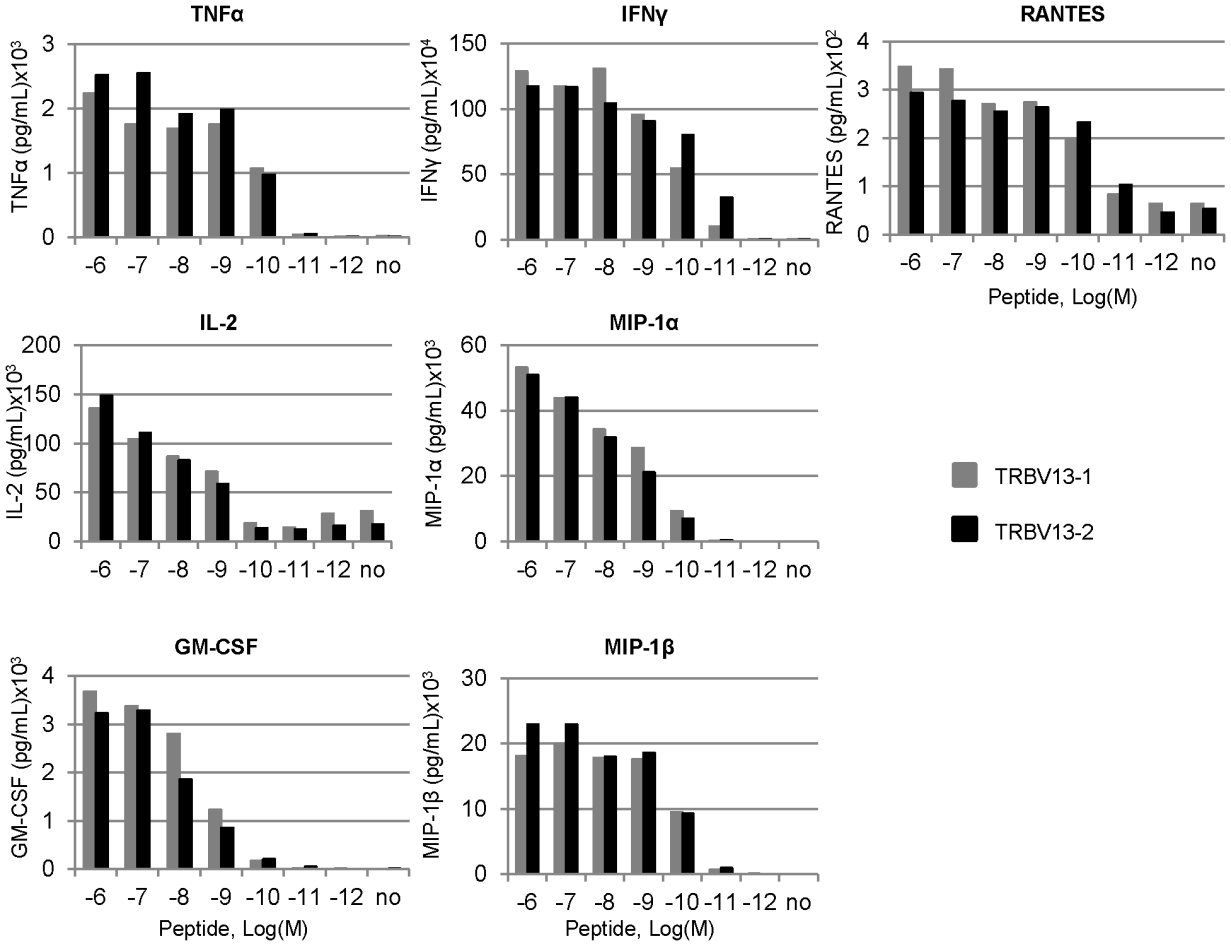
Cytokine production following 72 hour stimulation of TCR Tg cells. Cells were stimulated for 72 hours with the indicted concentration of peptide and cytokine concentrations in the supernatant were measured using ELISA. Data are representative of three independent experiments.

In order to evaluate cytokine production following stimulation of TCR Tg cells, we measured their cytokine production following 5 hour stimulation of naïve cells by intracellular cytokine staining (ICS) and analyzed cytokine secretion by ELISA following 3 days of stimulation (Figures 4 and 5, respectively). Following 5 hours of stimulation, TNFα was the predominant cytokine expressed by both TRBV13-1 and TRBV13-2 cells. TNFα production is a hallmark of the activation of naïve CD8+ T cells [24], and production of this cytokine was considerably higher than production of IL-2 or IFNγ after 5 hours of stimulation (Figure 4).

Analysis of cell culture supernatants after three days in culture revealed antigen and dose-specific production of TNFα, IFNγ, IL-2, MIP-1α, MIP-1β, GM-CSF, and RANTES, a profile of cytokine expression is typical of CD8+ T cells stimulated by cognate antigen (Figure 5). IFNγ, in particular, was produced in high amounts by both lines when cells were stimulated for three days, reflecting the transition between the naïve response of TNFα production to higher production of IFNγ. The threshold for production of IFNγ by both lines was 10^−11^ M peptide, while the threshold level for production of detectable levels of most cytokines ranged from 10^−9^ to 10^−10^ M (Figure 5).

### TRBV13-1 cells exhibit higher cytolytic capacity than TRBV13-2 cells

**Figure 6:**
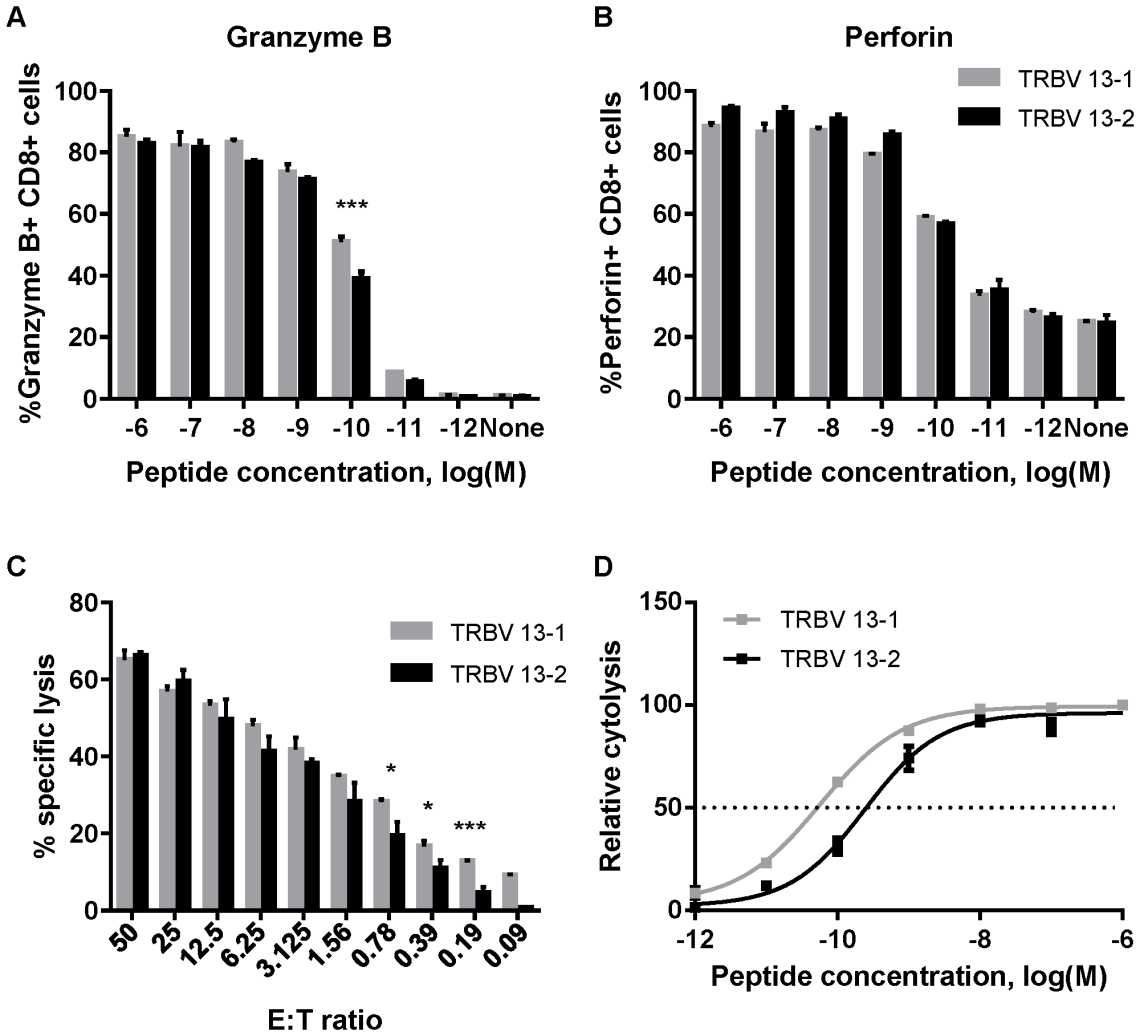
Cytolytic properties of TCR Tg cells. (**A and B**) Cells were stimulated for 24 hours in the presence of M2 peptide in graded concentrations. The percentage of CD3+CD8+ cells positive for Granzyme B (A) or Perforin (B) were determined by intracellular staining. (**C**) CFSE-labeled target cells were loaded with 10^−6^ M M2 peptide and cytolytic activity of Tg CD8+ cells was determined by *in vitro* killing assay at different E∶T ratios. (**D**) Target cells were labelled with peptide at graded concentrations and mixed with effector cells at E∶T ratio of 50. EC_50_ was determined by a 4 parameters non-linear fit. *p≤0.05, ***p≤0.001 and error bars represent the SEM.

Cytolytic activity is a main characteristic of activated CD8+ T cells. In order to compare the cytolytic capacity of our two TCR Tg lines we measured cellular mediators of cytotoxic function [25]. Naïve cells were stimulated for 24 hours as described above and stained for Granzyme B (Figure 6A) and perforin (Figure 6B) as markers for degranulation. Both cell lines exhibited Granzyme B and perforin staining in a dose-dependent manner to M2 peptide concentration. More than 80% of cells were positive for both Granzyme B and perforin in peptide concentrations of 10^−6^ to 10^−9^ M and about 50% of cells degranulated at M2 peptide concentration of 10^−10^ M. To further evaluate the cytotoxicity of the two M2-specific Tg lines, we measured their direct cytolytic acitivity by *in vitro* CTL assay. Target cells were pulsed with M2 peptide at different concentrations before they were mixed with activated isolated CD8+ cells (see materials and methods). At effector to target (E∶T) ratio 1.56 to 50, no statistical difference was apparent between the cytotoxicity capacity of the two Tg cell lines. However, at E∶T ratio of 0.78 or lower, TRBV13-1 cells exhibited significantly higher cytolysis than TRBV13-2 cells (Figure 6C, P<0.05). Additionally, we compared the cytolytic activity of the two cell lines in response to different concentrations of M2 peptide (Figure 6D). TRBV13-1 exhibited cytolytic activity in response to a significantly lower M2 peptide concentration than TRBV13-2 (EC_50_ = 5.71×10^−11^ M and 2.43×10^−10^ M, respectively).

## Discussion

We present here the first TCR Tg mice specific for the dominant K^d^M2_82–90_ RSV epitope of RSV. These mice were designed by a unique methodology. Single-cell clonotyping was performed on epitope-specific, tetramer-sorted populations of cells from RSV-infected mice and individual TCR sequences were chosen based on specific features of the CDR3β sequences [20], [26]. To the best of our knowledge this is the first time that direct *ex vivo* sequencing has been exploited to select clones rather than using typical methods of *in vitro* enrichment and functional cloning. The main advantage of this approach is that avoiding *in vitro* competition and/or manipulation reduces selection bias and may be more representative of the physiological response. This method requires a deeper understanding of the polyclonal response to a specific epitope, and selection of clones with common or unique features from a larger pool of sequences. The TRBV13-2 clone was selected because the majority of cells specific for the K^d^M2_82–90_ epitope express TRBV13-2, and the CDR3β sequence was common among infected mice (public). The TRBV13-1 TCR Tg expresses a more private, rare CDR3β response to the same epitope. This study represents a comprehensive comparison of the functional profile of these two novel TCR transgenic strains and is the first example of two different TCR Tg lines established toward the same epitope with distinct features.

One of the remarkable features of the CD8+ T cell response in RSV-infected BALB/c and CB6F1 mice is the numeric dominance of the M2-specific response over the response to all other viral epitopes. Our proliferation data indicates that M2-specific CD8+T cells are highly proliferative *in vitro* and *in vivo* in infected animals. The numerical dominance of the M2-specific response may be a combined effect of early activation, the highly proliferative nature of T cells for this epitope, and a potential doubling time of less than 6 hours for activated cells *in vivo* [27], [28].

Despite sharing the same specificity and a similar intrinsic affinity for MHC-peptide, TRBV13.1 and TRBV13.2 TCR Tg lines showed some functional differences, primarily at threshold peptide concentrations. TRBV13.1 cells tended to proliferate better at lower peptide concentrations *in vitro* (Figure 2B), and synthesized DNA faster post-stimulation than TRBV13.2 cells at a high peptide concentration (Figure 2C). In infected mice, however, TRBV13-2 cells proliferated better than TRBV13-1 (Figure 3). We also found that TRBV13-1 cells had higher cytolytic activity than TRBV13-2 cells at threshold peptide concentrations (Figure 6).

Two elegant studies have demonstrated that CD8+ T cells have an astounding functional and phenotypic diversity [29], and that they can evolve with time and exhibit programmatic trajectories of cytokine secretion [30]. Among our new RSV-specific TCR Tg lines, we found that the “built-in” profile of each TCR clone was retained through a large range of peptide concentrations. Both TRBV13-1 and TRBV13-2 proliferate readily and express proinflammatory cytokines at peptide concentrations higher than 10^−11^ M. Factors dictating *in vivo* CD8+ T cell function can be more complex, where peptide presentation and other inflammatory signals produced in local infected microenvironments may have a profound influence. Studies on the functional hierarchy of CD4+ T cells have supported a model in which different effector functions have a hierarchical order of elicitation thresholds [31]. Based on proliferation, cytokine production and cytolytic capacity, each T cell may have a unique hierarchy of functions. Studies in many labs as well as our own suggest that the synthesis of TCR-mediated signals and signals from costimulatory molecules and the inflammatory milieu may alter activation thresholds of individual functions and dictate the behavior of CD8+ T cells [32]–[34]. Future structure-function studies may reveal how TCR-mediated interactions fine-tune the phenotype and character of each response. By studying two transgenic clones specific for the same epitope in parallel, we can also address how the strength and duration of TCR-mediated signaling regulates downstream effects and give further insight into how even subtle differences in structure may dictate the outcome of clonal responses.

This is the first time two distinct TCR Tg mice with T cells that can be detected by the same class I tetramer have been generated. While both M2-specific lines have modestly different functional properties, the overall characteristics of both reflect the dominant traits of the M2-specific polyclonal response. To fully recapitulate a polyclonal T cell response, it may be necessary to combine the responses of dozens or hundreds of individual epitope-specific cells. These novel TCR Tg mice provide a tool not only for basic RSV research, but also for fundamental studies investigating TCR structure-function relationships and the induction of CD8+ T cell functional responses.
